# Predictive factors for foot pain in the adult population

**DOI:** 10.1186/s12891-023-07144-9

**Published:** 2024-01-12

**Authors:** Francisco Javier Canca-Sanchez, Jose Miguel Morales-Asencio, Ana Belen Ortega-Avila, Gabriel Gijon-Nogueron, Pablo Cervera-Garvi, Ana Marchena-Rodriguez, Jose Carlos Canca-Sanchez

**Affiliations:** 1 Faculty of Health Sciences, Department of Nursing and Podiatry, University of Malaga, Málaga, Spain; 2 Instituto de Investigación Biomédica de Málaga y Plataforma en Nanomedicina-IBIMA platform BIONAND, Málaga, Spain

**Keywords:** Foot, Pain, Predictive, Factors, Volume

## Abstract

**Background:**

Foot pain has been associated to factors like: fat, body mass index, age increased, female gender and the presence of pathologies. Although evidence is limited. The purpose is to determine the predictive factors for foot pain in the adult population.

**Methods:**

From January to December 2021, 457 patients were > 18 years, gave signed informed consent to take part to this cross sectional study. All completed demographic data and various questionnaires related to pain: Foot Function Index, EuroQoL-5D and Visual Analogue Scale (foot pain). Anthropometric measurements were obtained using McPoil platform and foot posture was assessed by the Foot Posture Index (FPI). To determine whether a volume change is a predictive factor for foot pain, a parameter was established: the volumetric index for footwear (VIF). Factors linked to the presence of pain, including the considered VIF variables, were analyzed through multivariable logistic regression.

**Results:**

Among the study population, 40.7% were male and 59.3% female. The mean age of 39.06 years and a body mass index of 25.58 Kg/cm^2^. The logistic regression model had a classification capability of 72.4%, a sensitivity of 72.3% and a specificity of 73%, in which, the predictors considered were the variables found to have a significant association with FFI-pain > 45 points,, showed that younger women, with a higher BMI, higher values of right FPI (pronation), poorer overall perceived health and with problems in walking were more likely to experience foot pain.

**Conclusion:**

Predictive factors for foot pain in the adult population include gender, age, Body Mass Index, FPI on the right foot, perceived health and mobility. Clinical implication, the presented measure aids physicians in assessing their patients´ foot pain likelihood.

## Background

Worldwide, pain is a major health problem [1], affecting 20% of all adults. Among the general population, foot pain is the most common variant, with an estimated prevalence of 13–36% [2]. Women are more likely than men to suffer this condition [3].

Foot pain is a risk factor for locomotor disability [4], impaired balance [5], increased risk of falls [6], loss of independence and reduced quality of life [7]. It has been associated to factors like: fat, body mass index [8] age increased and female gender [9]. The presence of metatarsalgia [10] and/or structural alterations (flat feet [11], hallux valgus [12, 13]), a range of psychosocial factors including depression and anxiety as well as reduced health-related quality of life (HRQoL) [14] and poorly-fitting footwear [15]. Whether tight or loose, inadequate footwear can increase plantar pressures and friction and may provoke foot ulcers [16], possibly leading to infection and even amputation [17]. To help avoid these outcomes, appropriate footwear [18] must be worn while the wearer is standing upright [19]. Ideally, it should be wide enough around the heel, made of leather, have no internal seams, present a semi-rigid buttress, with a wide anatomical sole, enable adjustment with laces or Velcro, and have a heel that is 2–3 cm high, providing cushioning, width and volume appropriate to foot size. Among others, Chen et al. [20] have shown that the volume of the foot and leg varies during sporting activity, when changing the resting position (from sitting to standing), when remaining in a standing position for an extended period [21] or even according to the time of day [22], and also with pathologies like plantar fasciitis [23] or ankle ligament injuries [24]. This change in volume can result in swelling, discomfort, blood pooling and muscle fatigue [25]. Although studies have been carried out on foot pain and footwear [16, 26], to our knowledge, no studies have evaluated whether a volumetric change in the foot within the shoe is a predictive factor for foot pain in the absence of previous pathology such as arthritis [27] or osteoarthritis or endocrine disorders such as diabetes mellitus, or, conversely, whether the most significant predictive factors in this respect are psychological factors, body mass index, gender and age [9, 14, 28]. There is evidence regarding predictive factors for chronic pain in the foot and ankle among populations in South Australia [29] and Tasmania [30]. However, this evidence does not include volumetric changes in the foot. Therefore, the purpose of this research is to identify predictive factors for foot pain in the adult population.

## Methods

### Ethical approval

This study received ethical approval from the Biomedical Research Ethics Portal (PEIBA) of the Regional Government. It was carried out in full accordance with the provisions of the Declaration of Helsinki regarding ethical principles for medical research involving human subjects.

### Design

Cross-sectional study.

### Study population

The participants were recruited at Podiatry Care Unit at the University of Malaga and private podiatry clinics in Malaga (Spain) from January to December 2021. All were > 18 years old, healthy, enjoyed cognitive and physical autonomy and with or without foot pain. All who expressed interest provided signed consent prior to the interviews and those who agreed to participate were given further details of the study.

The exclusion criteria applied were the presence of musculoskeletal, vascular or peripheral nervous system disease, or endocrine disorder (especially diabetes mellitus), or smoking. Also excluded were any persons who had undergone a surgical intervention on the foot, who wore orthoses or who had received any other orthopaedic treatment.

### Procedures

Study data were compiled by a member of the research team in a two-phase operation. In the first, each participant was asked to complete a form providing demographic data (age, gender, height, weight, shoe size) and to respond to various questionnaires related to pain: the Visual Analogue Scale (VAS) for foot pain [31] and the Spanish-language versions of the EuroQoL-5D questionnaire [32] and the Foot Function Index (FFI) [33]. The EuroQoL-5D is a self-administered questionnaire that assesses health status in terms of five specific dimensions (mobility, personal care, daily activities, pain/discomfort and anxiety/depression). The VAS is more general [34]. The FFI is also self-administered. It is a valid and reliable instrument, with an intraclass correlation coefficient (ICC) of 0.87 and a Cronbach’s alpha of 0.96. This questionnaire is composed of three subscales: pain, disability and limitation of activity, with ICC values of 0.69, 0.81 and 0.84 and Cronbach’s alpha of 0.95, 0.93 and 0.73, respectively [35].

In the second phase, and using the foot measurement platform described by McPoil [36, 37], one of the researchers conducted a repeatability and reliability analysis of this procedure, with 30 participants (ICC for the instrument, 0.96–0.98), focusing on the following anthropometric parameters: midfoot and forefoot length; hindfoot width; midfoot height (load-bearing and non-load-bearing). Each participant was asked to stand on the platform to obtain the load-bearing measurement and then to sit down for the non-load-bearing measurement [38]. In both cases, the body weight was evenly distributed between the 2 feet. For the load-bearing measurement, the patient was instructed to stand in a relaxed position, looking straight ahead. The measurements were taken with the patient’s feet in the heel cups of the measurement platform, with the heels as far back as possible, and with the first metatarsal heads against the surface boundary. The same measurements were then obtained with the participant in a seated position on the platform (non-load-bearing). Foot posture was evaluated using the Foot Posture Index (FPI) (ICC for the clinician, 0.94–0.96) [39]. Each criterion was scored as − 2, − 1, + 1 or + 2. The following FPI cut-off points, defining foot type category, were used: a) -12 to − 4 = highly supinated; b) -3 to 0 = supinated; c) + 1 to + 6 = neutral; d) + 7 to + 10 = pronated; e) + 11 to + 12 = highly pronated [40].

### Sample size

The sample size established was sufficient to enable minimum correlation coefficients of 0.15 with an alpha of 0.05. Calculation showed this value to be 346 participants, for a two-tailed hypothesis test. This size was then increased by 3% to compensate for possible losses or invalid data. In total, thus, a sample size of 449 participants was required to perform the correlation analysis.

For the regression analysis, the Peduzzi et al. [41] rule was applied to determine the necessary sample size. For a maximum of four possible predictors and taking the less optimistic prevalence of foot pain estimated by Gates et al. [2], we calculated that 308 participants would be required. 

### Statistical analysis

Descriptive statistics of the variables were obtained and the normality of their distribution was confirmed by the Kolmogorov-Smirnov test. The mean differences between independent groups were analysed by bivariate analysis: Student’s t test for normally-distributed variables and the Mann-Whitney U test otherwise. In addition, bivariate analyses of quantitative variables were performed, using Pearson correlations and Spearman’s rho, depending on the normality of the distributions. Bivariate analyses for the qualitative variables were also performed using the chi-square test or Fisher’s exact test when necessary.

A key parameter in determining the predictive factors for foot pain is that of the volumetric index for footwear (VIF). This index is constructed as follows: (right foot length_X * ((right hindfoot width_Z1 + right midfoot width_Z2 + right forefoot width_Z3) / 3) * (maximum height of right internal longitudinal arch_Y1 + maximum height of right first metatarsophalangeal joint_Y2) / 2)) / 1000).

$$VIF=\frac{\left.\textrm{right}\ {\textrm{foot}\ \textrm{length}}_{\textrm{X}}\ast \left(\left(\textrm{right}\ \textrm{hindfoot}\ \textrm{widt}{\textrm{h}}\_\textrm{Z}1+\textrm{right}\ \textrm{midfoot}\ \textrm{widt}{\textrm{h}}\_\textrm{Z}2+\textrm{right}\ \textrm{forefoot}\ \textrm{widt}{\textrm{h}}\_\textrm{Z}3\right)/3\right)\ast \left(\textrm{maximum}\ \textrm{height}\ \textrm{of}\ \textrm{right}\ \textrm{internal}\ \textrm{longitudinal}\ \textrm{arc}{\textrm{h}}\_\textrm{Y}1+\textrm{maximum}\ \textrm{height}\ \textrm{of}\ \textrm{right}\ \textrm{first}\ \textrm{metatarsophalangeal}\ \textrm{join}{\textrm{t}}\_\textrm{Y}2\right)/2\right)\Big)}{1000}$$

The association between the predictive factors of adequacy of shoe size, namely the VIF obtained for a load-bearing stance and the other clinical and sociodemographic characteristics considered, was determined by means of multivariate linear regression, taking shoe size as the dependent variable and the variables found to be significant in the bivariate analysis as the predictors. The assumptions of linearity, independence, homoscedasticity, residual normality and collinearity were tested by residual analysis, the Durbin-Watson statistic, tolerance levels and the variance inflation factor. All confidence intervals were calculated at 95%.

The factors associated with the presence of pain, including the volumetric index variables considered, were analysed by multivariable logistic regression, following intentional selection and the univariate analysis of each variable. In this iterative selection process, variables were eliminated from the model if they were not significant and did not act as a confounding factor. Statistical significance was assumed at the 0.05 alpha level and a confounding factor was defined as a change > 20% in any remaining parameter compared to the full model. The model fit always included the variables age and gender, together with those estimated in the model. The overall fit to the rule was evaluated by Nagelkerke’s R^2^ parameter, adjusted by degrees of freedom to avoid estimates of overfitted models, and by the Hosmer and Lemeshow test. In addition, the − 2 log likelihood statistic (−2LL) was calculated and the class separation capacity of the model was estimated by the ROC curve. In the statistical modeling, it was not necessary to address the issue of missing data, and no imputations were required.

All statistical analyses were performed with SPSS v.26 and JAMOVI 2.2.

## Results

Among the 457 participants, 40.7% were men and 59.3% women, with an average age of 39.06 years and BMI of 25.58 Kg/cm^2^. The shoe size worn ranged from 37 to 47 (median 40 (IQR: 5) and the median right and left FPI values were 6 (IQR: 2) and 6 (IQR: 1), respectively. The prevalence of foot pain in the participants was 36%. The following median pain measures were obtained: EuroQoL-5D-VAS-General 2 (IQR: 5) and VAS-f 3 (IQR: 6); the mean value obtained for the FFI-pain subscale was 22.52 (SD: 19.33). The median VIF values for the right foot (load bearing vs. non-load bearing) were 884.58 (IQR: 328.07) and 898.77 (IQR: 337.45), respectively. The corresponding values for the left foot were 908.06 (IQR: 321.77) and 927.88 (IQR: 321.61). The characteristics of the sample population are described in detail (Table 1).

**Table 1:** General characteristics of the population

	Male *N* = (186) Median (IQR)	Female *N* = (271) Median (IQR)	p
***Age***	39.5 (19.8)	35.0 (25.0)	0.015
***BMI***	25.9 (4.18)	24.1(5)	< 0.001
***Shoe size***	43.0 (3.00)	38.0 (2.00)	< 0.001
***EuroQoL-VAS_Gen***	2.00 (4.00)	3.00 (5.00)	0.039
***VAS_Feet***	2.00 (5.00)	5.00 (4.00)	< 0.001
***FFI_Pain***	13.5 (27.0)	26.0 (33.0)	< 0.001
***VIF_LB_Right***	1103 (242)	774 (143)	< .001
***VIF_LB_Left***	1140 (253)	789 (160)	< .001
***VIF _NLB_Right***	1135 (252)	792 (147)	< .001
***VIF _NLB_Left***	1186 (235)	804 (165)	< .001
***diff_LB/NLB_VIF_R***	−16.7 (65.2)	−10.1 (47.6)	0.206
***diff_ LB/NLB_VIF _L***	−23.6 (73.3)	−12.8 (42.7)	0.011
***diff_LB_Left-Right_VIF***	−29.0 (67.1)	−19.3 (50.2)	0.055

*BMI* Body mass index, *EuroQoL-VAS--Gen* EuroQoL- Visual Analogue Scale- General, *VAS* Visual Analogue Scale, *FFI* Foot function index, *VIF* Volumetric index for footwear, *diff* Difference, *LB* Load bearing, *NLB* Non-load bearing

**Fig. 1:**
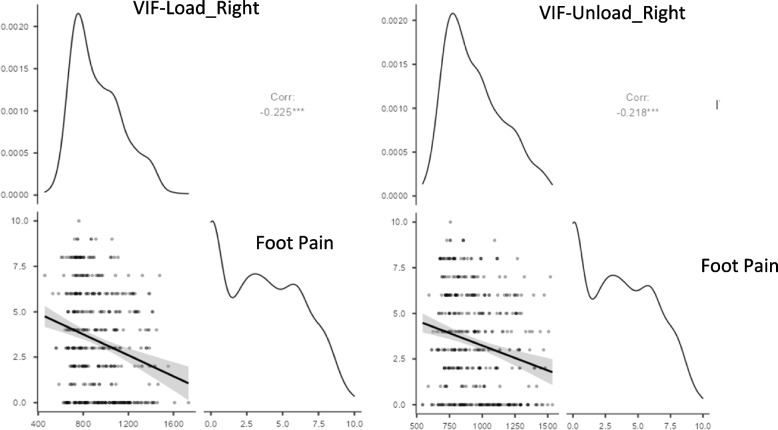
The correlacion between Volumetric index for footwear_Right and foot pain. VIF: Volumetric index for footwear

**Fig. 2:**
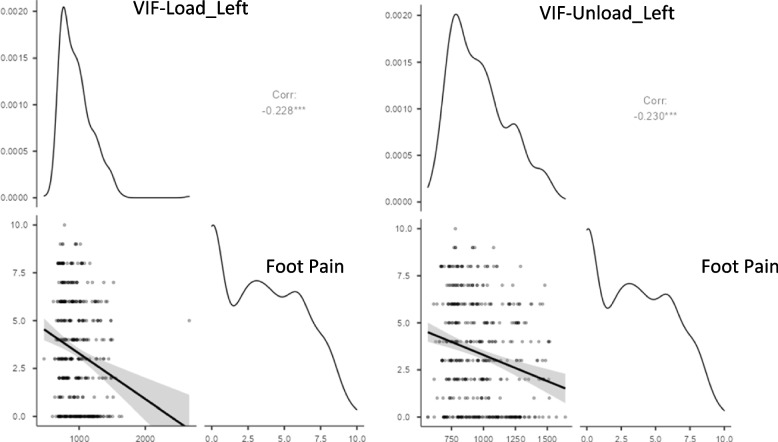
The correlacion between Volumetric index for footwear_Left and foot pain. VIF: Volumetric index for footwear

Statistically significant weak inverse correlations were found between shoe size and both VAS-f (rho = − 0.18; *p* < 0.01) and the FFI-pain subscale (rho = − 0.21; *p* < 0.01). The correlation between VIF and shoe size was strong and statistically significant, in both feet, load bearing and non-load bearing (*p* < 0.001), while between VIF and foot pain it was inverse and significant (*p* < 0.001) (Figs. 1 and 2).

Finally, we considered the possible association between the study variables and the level of foot pain. To do so, the FFI-pain subscale was divided into two sectors, ≥45 points (corresponding to 13.8% of the participants) and < 45 points (86.2% of participants). The logistic regression model, in which the predictors considered were the variables found to have a significant association with FFI-pain > 45 points, showed that younger women, with a higher BMI, higher values of right FPI (pronation), poorer overall perceived health and with problems in walking were more likely to experience foot pain. This model had a correct classification capacity of 72.4%, with a sensitivity of 72.3% and a specificity of 73% for a cut-off point of 0.12, and an area under the curve of 0.83 (Table 2). In this model, the volumetric variables of the study did not reveal a significant association in the presence of other, more robust, predictors, such as perceived health.

**Table 2:** Predictor Factors

Predictor	Estimator	p	OR	Lower	Upper
Constant	−2.6409	0.102	0.0713	0.00302	1.683
Gender:					
Female – Male	1.5043	< .001	4.5010	2.11446	9.581
Age	−0.0467	< .001	0.9544	0.92964	0.980
BMI	0.1038	0.013	1.1093	1.02230	1.204
FPI_TOTALRight	0.1679	0.040	1.1828	1.00784	1.388
EuroQoL_VAS	−0.0393	< .001	0.9615	0.94175	0.982
EuroQoL_Mov:					
Difficulty walking – No difficulty walking	1.7515	< .001	5.7631	2.80954	11.821

Nagelkerke’s R^2^: 0.33

*BMI* Body Mass Index, *FPI_Total_Right* Foot Posture Index_Total_Right, *EuroQoL_VAS* EuroQoL_Visual Analogue Scale, *EuroQoL_Mov* EuroQoL_Movement

## Discussion

Our study aim was to determine whether the predictive factors for foot pain in the adult population include psychological factors, body mass index, gender and age or if a volumetric change in the foot within the shoe is the most significant predictive factor for foot pain.

Our findings showed the following predictive factors for foot pain (FFI-pain > 45 points): gender (specifically female), age, BMI, right FPI (high values for pronation), perceived general health and mobility (difficulty in walking). The logistic regression model obtained a correct classification of 72.4%, with a sensitivity of 72.3% and a specificity of 73% for a cut-off point of 0.12, and an area under the curve of 0.83. On the other hand, neither the VIF nor shoe size were predictive factors, which contrasts with some previous investigations according to which correctly-fitting footwear offers physical protection, acting as an environmental barrier for the foot, providing biomechanical support and enabling pressure redistribution across the foot, thus reducing the formation of calluses and therefore foot pain [42, 43].

Our study corroborates previous research findings that female gender is not only associated with increased exposure to pain [44, 45], but also that it is a predictive factor of foot pain, compared to male gender. This could be due to the structural differences that exist between men and women, since the latter more frequently present pathologies in different anatomical regions [46]. Moreover, in general women more frequently evaluate their health and seek the necessary treatment. In most cases, the gender difference is not associated with any specific age [47]. BMI is a risk factor for various pathologies, including plantar fasciitis [48, 49], foot pain [50] and also depressive symptoms due to perceptions of inadequate weight. The latter impact is especially associated with female gender [14, 51]. Gate et al., 2020 [2] showed foot pain to be more prevalent in women and obese individuals and it generally increased with age, with the prevalence being much lower in younger participants (20–44 years). However, our results differ, as being woman is considered a predictive factor for foot pain. Regarding BMI, it has been considered that a high BMI is believed to be associated with pronated feet, but our results showed that the highest levels of BMI were associated with a neutral foot position. From this, we conclude that there is no direct relationship, and even that a pronated foot position may be associated with male gender in young, healthy subjects, as reported by Gonçalves de Carvalho et al. [52]. However, abnormalities in foot posture (pronated or supinated) do change the distribution of forces in the lower leg, and pathologies may develop not only in the foot (such as heel pain) but may also affect higher structures, for example provoking osteoarthritis of the knee, and these abnormalities are usually associated with pronated feet [53].

The main limitation of the study findings is the composition of the study sample, which predominantly consisted of female patients, persons with a neutral foot posture and the interior volume of the footwear was not taken into consideration. Anthropometric measurements of the foot could only be taken in a static (loaded and unloaded) position. They should also be measured during walking to ensure that there are no changes in these measurements. However, the study also presents important strengths, namely the methodological rigour of the protocol used. Secondly, regarding the anthropometric measurements procedure, we obtained an ICC range of 0.96–0.98 for the instrument. This not only highlights the precision of our measurements but also bolsters the credibility of our findings. The meticulous attention to repeatability and reliability ensures the validity of our study outcomes and significantly contributes to the overall robustness of our research. Furthermore, to our knowledge, this is the first investigation in this field to take account of the VIF among the characteristics that constitute predictive factors of foot pain. Future research should be undertaken to address specific population groups such as those with diabetes mellitus, fibromyalgia or rheumatoid arthritis or those who practice particular sports. In addition, a larger number of supinated or pronated foot categories should be included in future study groups.

The present study has important clinical implications, as the VIF serves as a measure that aids physicians in assessing the likelihood of their patients suffering foot pain. The measure achieves a sensitivity of 72.3% and is very straightforward to apply, offering and providing practical benefits to both clinicians and researchers. This advancemet enhances both pain prevention and patient treatment approaches.

## Conclusion

The findings showed the most significant predictive factors for foot pain in the adult population which include female gender, age (younger women), BMI (higher BMI), right foot FPI (higher values of pronation), perceived health, and mobility (poorer overall perceived health and with problems in walking).

## Data Availability

All data generated or anlysed during this study are included in this publised article.
